# C-Terminus of Ca_v_1.3 L-Type Ca^2+^ Channel Upregulates Its Own Gene Expression

**DOI:** 10.3390/cells15090828

**Published:** 2026-05-01

**Authors:** Yvonne Sleiman, Ujala Srivastava, Jean-Baptiste Reisqs, Raj Wadgaonkar, Yongxia Sarah Qu, Valérie Pouliot, Mohamed Chahine, Mohamed Boutjdir

**Affiliations:** 1 Cardiovascular Research Program, VA New York Harbor Healthcare System, New York, NY 11209, USA; yvonne_sleiman@hotmail.com (Y.S.); ujalasrivastava@googlemail.com (U.S.); jeanbaptiste.reisqs@gmail.com (J.-B.R.); qu.sarah@gmail.com (Y.S.Q.); 2 Department of Medicine, Cell Biology and Pharmacology, State University of New York Downstate Health Sciences University, New York, NY 11203, USA; 3 Department of Medicine and Cell Biology, College of Medicine, State University of New York Downstate Health Sciences University, Brooklyn, NY 11203, USA; raj.wadgaonkar@downstate.edu; 4 Department of Cardiology, New York Presbyterian Brooklyn Methodist Hospital, New York, NY 11215, USA; 5 CERVO Brain Research Centre, University Institute of Mental Health of Quebec, Quebec City, QC G1E 0N5, Canada; valerie.pouliot@cervo.ulaval.ca (V.P.); mohamed.chahine@phc.ulaval.ca (M.C.); 6 Department of Medicine, Faculty of Medicine, Laval University, Quebec City, QC G1V 0A6, Canada; 7 Department of Medicine, New York University Grossman School of Medicine, New York, NY 10016, USA

**Keywords:** calcium channel, C-terminus, gene expression, nuclear translocation, Ca_v_1.3 upregulation

## Abstract

**Highlights:**

**What are the main findings?**

- Nuclear translocation of Ca_v_1.3-C-terminus leads to upregulation of Ca_v_1.3 transcription.
- The Ca_v_1.3-C-terminus may regulate Ca_v_1.3 promoter activity, potentially involving MEF2-dependent mechanisms.

**What is the implication of the main finding?**

- Harnessing the intrinsic properties of the C-terminus as a trans-regulator of its own gene expression may represent a potential strategy to modulate cardiac function.

**Abstract:**

The Ca_v_1.3 L-type calcium (Ca^2+^) channel plays a critical role in cardiac excitation-contraction coupling, regulating heart rate, contractility, and gene expression. The C-terminus of Ca_v_1.3 has recently been shown to translocate to the nucleus and act as a transcriptional factor to modulate the function of Ca^2+^-activated K^+^ channels in atrial cardiomyocytes. However, the role of the Ca_v_1.3-C-terminus in the regulation of transcription of its own Ca_v_1.3 gene remains unknown. We evaluated the impact of the nuclear translocation of the Ca_v_1.3-C-terminus on the transcription of the Ca_v_1.3 gene and Ca_v_1.3 promoter activity in vitro using cultured neonate rat ventricular myocytes (NRVMs), and mouse atrial cardiomyocytes (HL-1). Lentiviral infection of NRVMs demonstrated that the cleaved Ca_v_1.3-C-terminus translocates to the nucleus where it acts as a trans-regulator. The C-terminus of Ca_v_1.3 increased transcription of Ca_v_1.3 in vitro in NRVMs and in vivo in mice ventricles. Additionally, MEF2 transcription factor binding sites within the Ca_v_1.3 promoter may contribute to the regulatory effect of the Ca_v_1.3-C-terminus. These data *are the first* to demonstrate unique upregulation of Ca_v_1.3 transcription by its own mobile Ca_v_1.3-C-terminus both in vitro and in vivo. These findings suggest that the Ca_v_1.3-C-terminus has intrinsic properties as a trans-regulator of gene expression and may contribute to the modulation of cardiac function.

## 1. Introduction

L-type calcium (Ca^2+^) channels play an important role in excitation-contraction coupling in cardiomyocytes by mediating Ca^2+^ influx into the cell [1,2,3]. Four isoforms of L-type Ca^2+^ channels exist: Ca_v_1.1, Ca_v_1.2, Ca_v_1.3 and Ca_v_1.4. While Ca_v_1.1 is found in the skeletal muscle, Ca_v_1.2 and Ca_v_1.3 are primarily expressed in the heart, neurons and endocrine cells and Ca_v_1.4 is expressed in retina/immune cells [3,4,5,6]. In the adult heart, the Ca_v_1.3 Ca^2+^ channel is exclusively expressed in the supraventricular tissue (Atria, sino-atrial node (SAN), and atrioventricular node), whereas Ca_v_1.2 is expressed in both the atrial and ventricular myocardium [7,8,9,10]. The pore-forming α_1D_ subunit of the Ca_v_1.3 L-type Ca^2+^ channel is encoded by the *CACNA1D* gene, while the pore-forming α_1c_ subunit of the Ca_v_1.2 L-type Ca^2+^ channel is encoded by the *CACNA1C* gene [2]. Functionally, Ca_v_1.3 activates at more negative membrane potentials of about −60 mV, whereas, Ca_v_1.2 activates at more depolarized membrane potentials at −40 mV [11]. Both the Ca_v_1.3 and Ca_v_1.2 channels are sensitive to dihydropyridines, which modulate their activity [12]. It has previously been reported that Ca_v_1.3 is involved in SAN automaticity, and Ca_v_1.3-knockout mice developed sinus bradycardia, various degrees of atrioventricular block, impaired Ca^2+^ homeostasis, and are susceptible to atrial fibrillation [7,8,9,13].

Beyond their canonical role as membrane ion channels, emerging evidence indicates that some L-type Ca^2+^ channels can also contribute to intracellular signaling through proteolytic cleavage of their C-terminal domains. This concept was first reported for Ca_v_1.2, where activity-dependent cleavage of the distal C-terminus generates a fragment capable of translocating to the nucleus and regulating gene transcription, referred to as the calcium channel-associated transcription regulator (CCAT) [14]. Subsequent studies have further implicated L-type Ca^2+^ channels in excitation-transcription coupling, linking membrane excitability to gene expression [15,16]. In addition to these transcriptional roles, the Ca_v_1.2-C-terminus has been shown to directly modulate channel gating and calmodulin-dependent signaling, highlighting its dual role in both membrane-associated and intracellular signaling processes [17]. Together, these findings support a broader paradigm in which L-type Ca^2+^ channels couple membrane electrical activity to downstream signaling and gene expression.

The C-terminus domain of Ca_v_1.3 has been implicated in the regulation of the Ca^2+^-activated potassium (K^+^) (SK2) channels [16]. In addition, alternative splicing within the C-terminus of Ca_v_1.3 regulates their electrophysiological properties, modulates their dihydropyridine sensitivity, and subsequently alters the Ca_v_1.3 channel’s function [18,19,20,21,22].

In line with the broader concept of channel-derived signaling, accumulating evidence supports a ‘Ca_v_1.3 cleavage hypothesis’, whereby the C-terminal region of Ca_v_1.3 undergoes proteolytic cleavage to generate distinct functional fragments. Biochemical studies have identified multiple Ca_v_1.3 protein forms, consistent with post-translational cleavage events that separate the distal C-terminus from the full-length channel [19,20,21,22]. Similar to Ca_v_1.2, the Ca_v_1.3 C-terminal fragment has been reported to translocate to the nucleus, suggesting a role in transcriptional regulation [16]. Importantly, proteolytic cleavage of Ca_v_1.3 may dynamically regulate channel gating properties, surface expression, and signaling pathways, indicating that the cleaved and full-length forms may serve complementary but distinct physiological functions. However, the molecular mechanisms governing Ca_v_1.3 C-terminal cleavage, nuclear translocation, and its specific transcriptional targets remain incompletely understood.

Consistent with the Ca_v_1.3 cleavage hypothesis, we have demonstrated that Ca_v_1.3 appears as two bands in Western blots, one at 250 kDa and the other at 190 kDa [10], corresponding to the full-length protein with the C-terminus (250 kDa) and the cleaved form lacking the distal C-terminus (190 kDa), respectively. While the Ca_v_1.3-C-terminus has been shown to translocate to the nucleus and act as a transcription regulator, modulating the activity of SK2 channels in atrial cardiomyocytes [16], no data are currently available on the role of the Ca_v_1.3-C-terminus in regulating the transcription of its own Ca_v_1.3 gene or other Ca^2+^ channel genes and the resulting potential therapeutic consequences. We have demonstrated that the Ca_v_1.3-C-terminus restored the ejection fraction and prevented arrhythmias in ischemia-induced murine heart failure [23]; however, the mechanism leading to increased channel activity and underlying these beneficial effects remains unclear. Here, we investigated the role of Ca_v_1.3-C-terminus nuclear translocation on regulating the transcription of Ca^2+^ channel genes and mapped key regions within the Ca_v_1.3 promoter that are critical for its regulation by the C-terminus.

Ca_v_1.3 expression is developmentally and tissue-specifically regulated in the heart, being broadly expressed in fetal and neonatal cardiomyocytes and largely restricted to supraventricular tissues in the adult heart [7,8,9,10]. This developmental shift provides a framework to investigate whether Ca_v_1.3 undergoes C-terminal cleavage and nuclear translocation in a physiologically regulated and context-dependent manner. To address this, we employed a combination of complementary cellular models. Neonatal rat ventricular myocytes (NRVMs) were used to assess Ca_v_1.3 cleavage in a developmental context where the channel is widely expressed. Adult rat atrial myocytes (ARAMs) were used to evaluate C-terminal cleavage and nuclear translocation in mature cardiomyocytes where Ca_v_1.3 expression is retained. Adult rat ventricular myocytes (ARVMs), in which Ca_v_1.3 expression is absent, served as a negative control to validate antibody specificity. The heterologous tsA201 cell system was used to examine Ca_v_1.3 localization and cleavage in the absence of cardiac-specific proteolytic activity. Finally, HL-1 atrial cardiomyocytes were used as a complementary cardiac-derived model to validate and extend findings in a controlled and reproducible system. To assess the potential of the Ca_v_1.3-C-terminus as a candidate for future gene therapy applications in cardiovascular diseases involving Ca^2+^ entry, we further employed an in vivo mouse model. Together, these models allowed us to systematically assess Ca_v_1.3 C-terminal cleavage and function across developmental stages and cellular contexts.

## 2. Materials and Methods

### 2.1. Ethical Statement

The VA NY Harbor Healthcare System’s Institutional Animal Care and Use Committee approved the protocol. Mice (C57BL/6N, wild-type) and rats (Sprague Dawley^®^) were maintained per The Guide for the Care and Use of Laboratory Animals (NIH 2011) and ARRIVE guidelines (2020). The animals were euthanized by anesthetic overdose (5% with precision-calibrated isoflurane vaporizer) and cervical dislocation.

### 2.2. Plasmids

The rat Ca_v_1.3-C-terminus, corresponding to amino acids 1906 to 2203 of the Ca_v_1.3 channel protein, was cloned into multiple cloning sites of Clontech’s lentiviral vector pLVX-GFP1-N1. This vector will be referred to as Ca_v_1.3-C-terminus (pLVX-GFP1_a1DCa_v_1.3). Packaging plasmid Pax2 and envelope plasmid pMD2.G were obtained from Addgene (Watertown, MA, USA) as a bacterial stab.

### 2.3. Lentiviral Infection–Lentivirus Assembly

HEK293 cells were cultured in a medium consisting of DMEM, 10% FBS and 2% penicillin/streptomycin at 37 °C and 5% CO_2_. The cells were then transfected with the following: 8 µg pLVX-GFP1_a1DCa_v_1.3, 4 µg pMD2G, and 8 µg Pax2 or the empty lentiviral vector pLVX-GFP1-N1 using Ca^2+^ phosphate transfection. The cell medium was then collected and filtered through a 0.45 µm PES filter. The filtrate was centrifuged in a Sorvall Ultra Pro 80 ultracentrifuge at 26,000 rpm for 2 h. The lentiviral precipitate was dissolved in 100 µL of PBS with Ca^2+^ and Mg^2+^ and was aliquoted and stored at −80 °C. The lentivirus titer was determined for further experiments in plaque-forming units (PFU) per mL.

### 2.4. Neonate Rat Ventricular Myocyte (NRVM) Culture

NRVMs were cultured from 1–2-day-old rat pups, which were decapitated before heart extraction from the opened chest cavity. The hearts were cut into quarters and the atria removed. The ventricles were placed into a solution of Hanks’ Balanced Salt solution (HBSS; without Ca^2+^ and Mg^2+^) and heparin and washed repeatedly. The heart pieces were then digested in 7 mL of a solution made of 100 mL HBSS and 60 mg trypsin from bovine pancreas (Sigma-Aldrich, St. Louis, MO, USA). Digestion was carried out in the solution for 7 min while stirring with a magnetic stirrer. After 7 min, the solution was collected and a further 7 mL of fresh HBSS/trypsin was again added to the heart pieces for an additional 7 min. This was repeated 6 times or until all of the heart tissue was digested and collected for centrifugation at 1500 rpm for 15 min. The resulting pellet was then dissolved in a complete medium consisting of DMEM, 10% FBS, and 2% Penicillin/Streptomycin and filtered through a 70 µm strainer. The filtered cells were then pre-plated for 1.5 h in 100 mm diameter cell culture dishes at 37 °C and 5% CO_2_. The medium was then collected and further plated onto 35 mm diameter cell culture plates for transduction. NRVMs were plated at 60–70% confluence, infected on day 2, and cultured for 48 h.

### 2.5. Mouse Atrial Myocytes (HL-1) Culture

The mouse atrial cardiomyocytes (HL-1) were cultured as previously described [24]. Briefly, HL-1 cells were maintained in Claycomb medium (Sigma-Aldrich, St. Louis, MO, USA) supplemented with 10% fetal bovine serum, 2 mM glutamine, 5% penicillin-streptomycin, and 0.1 mM norepinephrine. The medium was replaced approximately every 24 h. Cells were incubated at 37 °C in a humidified atmosphere with 5% CO_2_ at 95% relative humidity. Upon reaching confluence, the cell cultures were split at a 1:3 ratio. The cells were used between passage 10 and 20. The differentiation of HL-1 cells was confirmed by their morphological characteristic (typical cardiomyocyte-like phenotype), as evident in bright-field images, and by the presence of spontaneous contractions.

### 2.6. Isolation of Cardiac Myocytes

Adult rat atrial and ventricular myocytes were isolated using Langendorff-perfused hearts as previously described [10,25,26]. Hearts were perfused at 37 °C with a HEPES-buffered solution containing 1.5 mg/mL collagenase type B for 8 to 15 min and subsequently dispersed in a KB solution composed of (mmol/L): 70 K glutamate, 10 KH_2_PO_4_, 30 KCl, 1 MgCl_2_, 10 glucose, 20 taurine, and 10 HEPES. Neonatal rat ventricular myocytes were isolated using a chopping method combined with trypsin digestion as described [10]. The cells were cultured on coverslips in Dulbecco’s Modified Eagle Medium supplemented with 10% calf serum (Gibco, Thermo Fisher Scientific, Waltham, MA, USA) and 2% penicillin/streptomycin before being used for immunostaining and infection experiments.

### 2.7. Transfection of TsA201 Cells

tsA201 cell transfection with the full-length Ca_v_1.3 along with β2/α2δ subunits was achieved as previously published [27,28]. Briefly, tsA201 cells were maintained in high-glucose DMEM medium (Gibco, Thermo Fisher Scientific, Waltham, MA, USA) supplemented with 2 mM glutamine, 10% fetal bovine serum, 100 U/mL penicillin G, and 10 mg/mL streptomycin. Cells were incubated at 37 °C, 5% CO_2_ and in a humidified atmosphere. The tsA201 cells were then transfected using the Ca^2+^ phosphate method using 7 µg of EBO/CD8 plasmid, and co-transfected in a molar ratio of 1:1:1 with 7 µg each of rat α1D, rat β (β2a), and rabbit α2/δ (α2δ-1) cDNAs.

### 2.8. Transduction of NRVMs and HL-1 Cardiomyocyte Transfection

When NRVMs were ~70% confluent they were transduced at a multiplicity of infection (MOI) of 120. The volume of lentivirus to be used was calculated using the following equation: MOI = PFU/number of cells. Lipofectamine 3000 reagent (Invitrogen; Waltham, MA, USA) was used for the transfection of HL-1 cardiomyocytes. The complete medium and Claycomb medium used for NRVM and HL-1 culture, respectively, were replaced with a mixture of OptiMEM, lentivirus (pLVX-GFP1_a1DCa_v_1.3 or pLVX-GFP1-N1), and polybrene (Thermo Fisher Scientific; Waltham, MA, USA) at a working concentration of 10 µg/µL for NRVMs and a P3000 reagent for HL-1 cardiomyocytes. The NRVM culture was then incubated overnight and on day 2 the media were switched again to complete media. The cultures were then further incubated for 48 h at 37 °C and 5% CO_2_. DNA lipid complex was added to the HL-1 cardiomyocytes and the cells were incubated at 37 °C and 5% CO_2_ for 72 h. On day 5, after first transduction, the NRVMs and HL-1 cells were imaged on the Olympus FSX 100 (Olympus Corporation, Tokyo, Japan) in both phase and green fluorescent modes. For each volume of lentivirus used, ten images were captured. The number of green fluorescent cells in each image was counted using Image J automated counting software (NIH, version 1.54p). We reached approximately a 100% infection rate, confirming the effectiveness of this delivery method.

### 2.9. Ca_v_1.3-C-Terminus Adeno-Associated Virus 9 Mice Infection

Mice, both males and females, were injected intraperitoneally either with 3 × 10^11^ GC/mL Ca_v_1.3-C-terminus adeno-associated virus 9 (AAV9-Ca_v_1.3-C-terminus) or phosphate-buffered saline (PBS) vehicle group for 30 days. After which, the mice were sacrificed for biomolecular experiments.

### 2.10. Protein Extraction and Western Blot

Membrane, cytoplasmic and nuclear proteins were extracted as previously described [9,10,28,29,30,31]. Equal amounts of protein (50–100 µg) were resolved on 4–12% SDS polyacrylamide gels. Immunoblots were incubated overnight at 4 °C with 1:500 anti- Ca_v_1.3 antibody (II–III cytoplasmic loop, (Sigma-Aldrich, St. Louis, MO, USA)) and 1:200 anti-C-terminus custom-made antibody (Pacific Immunology, Ramona, CA, USA), developed with horseradish peroxidase-labeled conjugated anti-rabbit antibody, and detected by enhanced chemiluminescence (ECL) (Amersham, Cytiva, Marlborough, MA, USA). The protein band densities were quantified using ImageJ software (NIH, version 1.54p; https://imagej.net/ij/, accessed on 24 November 2025). The negative controls included pre-incubation of the anti-C-terminus antibody with its antigenic peptide. 

### 2.11. Indirect Immunofluorescence Staining

Immunofluorescent staining was performed as previously described [10,28,32]. Briefly, cardiac cells were fixed, permeabilized, blocked, and incubated overnight at 4 °C with a primary anti-C-terminus antibody (Pacific Immunology, Ramona, CA, USA) targeting a unique epitope (amino acids 1708–1724) in the C-terminus of Ca_v_1.3 and detected with FITC-conjugated anti-rabbit antibody (1:200). The cells were visualized using a confocal scanning laser microscope (MRC-600; Bio-Rad, Hercules, CA, USA) with an XYZ scan. A surface plot was generated to illustrate the staining pattern of the anti-Ca_v_1.3 antibody. The secondary antibody alone and anti-Ca_v_1.3 antibody pre-incubated with its antigenic peptide were used as negative controls.

### 2.12. Targeted Gene Expression Profiling

NRVMs infected with either the control (GFP-lentivirus alone) or Ca_v_1.3-C-terminus were harvested after 48 h, and total RNA was prepared using the SA Bioscience protocol (Qiagen, Hilden, North Rhine-Westphalia, Germany). The targeted gene expression profile was assessed and analyzed using RT^2^ Profiler PCR arrays. Pathway-focused PRC arrays for the rat ion channels/transporter pathway, rat G-protein coupled receptors, rat signal transduction pathway, and rat MAP kinase signaling pathway were selected for gene expression analysis [33]. Each array contains a panel of 96 or 384 primer sets, targeting 84 or 370 well-researched pathway-focused genes, along with 5 housekeeping genes and 3 RNA and PCR quality controls.

Reverse transcription control: Using the RT^2^ First Strand cDNA Synthesis Kit (C-03), reverse transcription efficiency was reported as ‘Pass’ if Delta Ct (AVG RTC–AVG PPC) ≤ 5; otherwise, it was reported as ‘Inquiry’.

Genomic DNA contamination control: With the same kit, genomic DNA contamination was considered absent if Ct (GDC) ≥ 35, with a ‘Pass’ reported. If Ct (GDC) < 35, it was reported as ‘Inquiry’. The results for the genes of interest were validated using an NRT when necessary.

### 2.13. RNA Isolation and RT-qPCR Analysis

Transduced NRVM cells [34] were collected and homogenized by vortexing in RLT buffer (Qiagen RNeasy mini kit; Hilden, Germany). Total RNA was purified using Oiagen’s RNeasy mini kit. RNA was quantified using Invitrogen’s Qubit 2.0 fluorometer (Thermo Fisher Scientific; Waltham, MA, USA). 1 µg of total RNA was then reverse-transcribed into cDNA using Applied Biosystem’s High-Capacity cDNA Reverse Transcription kit and qPCR was then carried out on cDNA using the TaqMan Fast Advanced Mastermix (Applied Biosystems; Waltham, MA, USA). Genes coding for rat Ca_v_1.3 (*CACNA1D*), Ca_v_1.2 (*CACNA1C*), and 18S were amplified on Applied Biosystem’s 7500 Real-Time PCR system. Taqman Gene Expression Assay primers were used and these were obtained from IDT (Integrated DNA Technologies; Coralville, IA, USA). Primers contained the double-quenched Probe (5’FAM/ZEN/3’IBFQ) and ROX passive reference dye was used.

### 2.14. Patch Clamp

The L-type Ca^2+^ currents were recordings in NRVM cells like previously described [35]. Briefly, patch clamp experiments were performed at room temperature using an Axopatch 200B amplifier (Axon Instruments, Foster City, CA, USA). For this experiment, the junction potential was always compensated and was smaller than 5 mV. Pipettes were made from borosilicate glass capillaries and fire-polished. The patch pipettes were filled with a solution containing (in mmol/L): 139.8 CsCl, 10 K-EGTA, 4 MgCl_2_, 0.062 CaCl_2_, 5 Na_2_-creatine phosphate, 10 HEPES, 3.1 Na_2_ATP, 0.42 NaGTP; pH was adjusted to 7.2 with KOH. The bath solution comprised (in mmol/L): 132 NaCl, 5.4 CsCl, 1.8 CaCl_2_, 1.8 MgCl_2_, 0.6 NaH_2_PO_4_, 5 4-amino-pyridine, 10 HEPES, 5 dextrose, 5 Na-pyruvate; pH was adjusted at 7.4. The L-type Ca^2+^ currents were recorded using 250 ms pulses from −40 mV to +60 mV with an increment of +10 mV.

### 2.15. Promoter Constructs

The vector backbone used for promoter cloning was Genecopoeia’s pEZX-PG02. This vector allows for the promoter region to be cloned upstream of the Gaussia luciferase gene. Full-length Ca_v_1.3 promoter DNA (1100 bp) was amplified and cloned into pEZX-PG02. Subsequently, the promoter deletion constructs were generated by cloning 995 bp, 635 bp, 540 bp and 86 bp of the promoter into pEZX-PG02. Figure 4 shows the Ca_v_1.3 promoter and the locations of the various primers used for amplification of N-terminal deletion constructs. Table 1 shows the primer pairs for *CACNA1D* promoter deletion constructs.

### 2.16. Transfection of NRVMs and HL-1 with Promoter Constructs

HL-1 cardiomyocytes and NRVMs were transfected with pEZX-PG02 containing Ca_v_1.3 promoter and promoter deletion constructs with lipofectamine 3000^®^ reagent (Invitrogen; Waltham, MA, USA). HL-1 cardiomyocytes and NRVMs were cultured to ~80% confluency in a 6-well plate and the DNA lipid complex was then added to HL-1 cardiomyocytes and NRVMs. Cells were incubated at 37 °C and 5% CO_2_ for 72 h. After 72 h the medium was collected and stored at −20 °C. RIPA buffer lysis was used to prepare the cell lysate which was stored at −20 °C.

### 2.17. Luciferase Assay

Luciferase assay was carried out using Secrete-Pair Gaussia Luciferase Assay Kit from GeneCopoeia (Rockville, MD, USA). We followed the protocol for high sensitivity using GeneCopoeia’s GL-H buffer. A volume of 100 μL of culture media collected from the different construct transfections (from wells containing approximately 500,000 cells per well) was added to the separate wells of a black 96-well plate. Buffer GL-H (10x) was diluted in water to make a 1× buffer. Substrate GL (10x) was then added to the diluted buffer GL-H. The mixture was then incubated for 25 min while capped and protected from light. After incubation, 100 µL of the reaction substrate was added to each well containing media. After another 30 s incubation, luminescence readings were taken using a Perkin Elmer Multimode Plate Reader Enspire model 2300 (Waltham, MA, USA). Protein concentration for each of the collected cell lysates was measured using the Qubit 2.0 fluorometer (Thermo Fisher Scientific; Waltham, MA, USA). RLU (Relative Luciferase/Luminescent units) values were normalized to the amount of protein in the respective well.

### 2.18. Statistical Analysis

Statistical analysis was conducted using GraphPad Prism software (version 9). The Shapiro-Wilk test was employed to assess data normality. Comparisons between two independent groups were analyzed using an unpaired t-test for parametric distributions and the Mann-Whitney test for nonparametric distributions. Comparisons between multiple independent groups were analyzed using ANOVA for parametric distributions. Results are shown as mean ± SEM. Statistical significance was defined as *p* < 0.05. All statistical parameters are provided in the corresponding figures and their legends.

## 3. Results

### 3.1. The Cleaved Form of the Ca_v_1.3-C-Terminus Is Localized Within the Nucleus

**Figure 1:**
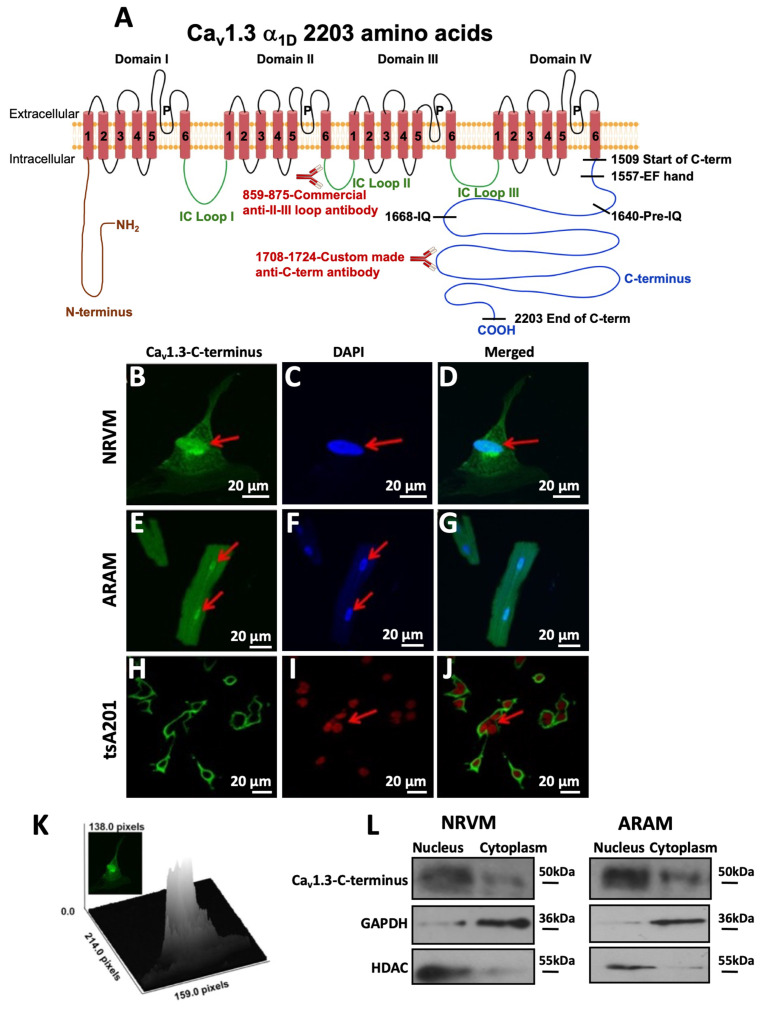
The cleaved form of the Cav1.3-C-terminus is localized within the nucleus. (**A**) Schematic representation of the Cav1.3 α1 subunit with the four domains (I–IV), six repeat segments (1–6), pore region (P), N-terminus (brown), intracellular loops (IC, green) and the C-terminus (C-term, blue) with the relevant regulatory domains (Isoleucine-glutamine (IQ); EF-Helix binding motif (EF hand)). The commercial anti-II-III loop and anti-C-terminus antibodies’ epitope are shown in red. Anti-Ca_v_1.3-C-terminus antibody labeling shows green fluorescent staining of the nucleus and the cytoplasm in (**B**) non-infected neonatal rat myocytes (NRVMs), (**E**) non-infected adult rat atrial myocytes (ARAMs) and (**H**) Ca_v_1.3-transfected tsA201 cells. DAPI in blue (**C**,**F**) and red (**I**) is a nuclear staining marker. Arrows indicate nuclear staining. Overlay images are shown in (**D**,**G**,**J**). The scale bar is 20 μm. (**K**) Surface plot illustrating the staining intensity of the C-terminus in the nucleus and cytoplasm using the anti-C-terminus antibody. The central gray peak corresponds to nuclear green fluorescence. (**L**) Representative Western blots of nuclear and cyptoplasmic fractions from non-infected NRVMs and ARAMs probed with the C-terminus antibody (n = 5). Histone Deacetylase (HDAC) and Glyceraldehyde 3-Phosphate Dehydrogenase (GAPDH) were used as internal controls.

To test whether the C-terminus of Ca_v_1.3 is cleaved under our experimental conditions, yielding a truncated channel and a cytoplasmic C-terminal fragment, we used an antibody to a unique epitope (amino acids 1708–1724) in the C-terminus (Figure 1A). This approach allowed to monitor the localization of the cleaved C-terminus. The specificity of the developed antibody was validated by comparison with a commercial antibody targeting the full-length protein. Unlike the commercial antibody, the developed antibody selectively recognized and immunoprecipitated only the short 50 kDa C-terminus fragment consistent with the predicted C-terminal fragment size (50 kDa) at the proteolytic site [36] (Figure S1A,B). The commercial Ca_v_1.3 antibody targeting the II-III cytoplasmic loop (amino acids 859–875, Figure 1A) detected a 190 kDa band corresponding to the truncated Ca_v_1.3 channel (short Ca_v_1.3) lacking the C-terminal fragment (Figure S1A), confirming cleavage of Ca_v_1.3. The full-length 250 kDa Ca_v_1.3 protein was not detected by either antibody, further supporting this observation. To further confirm the specificity of the developed antibody, we immunostained adult rat ventricular myocytes (ARVMs). The adult human ventricles do not express Ca_v_1.3 [37], and we have previously shown the absence of Ca_v_1.3 in the adult rat ventricle [10]. The absence of immunostaining in ARVMs (Figure S1C,D) demonstrates that the custom-made antibody used in this study does not recognize the C-terminus or any other region of Ca_v_1.2, confirming its selective specificity for the C-terminal fragment of Ca_v_1.3.

We next used confocal immunostaining to track the subcellular localization of the C-terminus of permeabilized non-infected neonatal rat ventricular myocytes (NRVMs) in culture, non-infected isolated adult rat atrial myocytes (ARAMs) and tsA201 cells. We found that the Ca_v_1.3-C-terminus antibody predominantly stained the nucleus and perinuclear region (marked by red arrows) and, to a lesser extent, the cytoplasm in both non-infected NRVMs (Figure 1B–D) and ARAMs (Figure 1E–G), suggesting that the C-terminus fragment is primarily localized in the nucleus. While we did not include T-tubule staining to distinguish atrial and ventricular cardiomyocytes, the lack of Ca_v_1.3 staining in ventricular rat myocytes in Figure S1C,D confirms the atrial origin of our cells presented in Figure 1E–G which showed cytoplasmic and nucleic staining with the Ca_v_1.3-C-terminus antibody.

The C-terminus of Ca_v_1.2, expressed in tsA201cells, is known to remain uncleaved and associated with the plasma membrane [38]. To further confirm the specificity of the Ca_v_1.3-C-terminus’s nuclear staining, we investigated whether the C-terminus of Ca_v_1.3, when expressed in tsA20 cells, remains associated with the plasma membrane. In tsA201 cells transfected with the full-length Ca_v_1.3 along with the β2/α2δ subunits required for Ca_v_1.3 expression [28,39,40], only surface membrane staining was observed, with no detectable nuclear staining (Figure 1H–J). This suggests that in tsA201 cells, the Ca_v_1.3-C-terminus either remains associated with the plasma membrane or Ca_v_1.3 is not cleaved. This further suggests that the nuclear staining observed in cardiac myocytes using the Ca_v_1.3-C-terminus antibody is specific and that the absence of Ca_v_1.3 cleavage in tsA201 cells confirms this distinctive characteristic of the native myocyte. To further confirm Ca_v_1.3-C-terminal cleavage, ARAMs were immunostained using the commercial antibody directed against a non-C-terminal epitope (loop II-III, Figure 1A). This antibody produced no detectable nuclear localization (Figure S1E,F). The absence of nuclear immunoreactivity for this non-C-terminal region supports the conclusion that the Ca_v_1.3-C-terminus is mobile and translocates to the nucleus. To provide a clearer representation of the nuclear staining intensity by the Ca_v_1.3-C-terminus antibody, a surface plot analysis was conducted on the same cell depicted in Figure 1B. A surface plot is a three-dimensional visualization of staining intensity within a cell. As shown in Figure 1K, the prominent nuclear staining is represented by a larger gray peak at the center of the cell, in contrast to the weaker staining observed around the nucleus and in the cytoplasmic regions. Taken together, these results support the conclusion that the C-terminus of Ca_v_1.3 undergoes cleavage and localizes to the nucleus in the native myocyte.

To further validate the presence of the C-terminus in the nucleus of cardiac myocytes, we investigated whether the nuclear labeling of the Ca_v_1.3-C-terminus corresponded to the 50 kDa C-terminus fragment, as we previously demonstrated [10]. To this end, we fractionated cultured NRVMs and ARAMs into nuclear and cytoplasmic compartments, followed by electrophoresis and blotting with the Ca_v_1.3-C-terminus antibody. The 50 kDa band mentioned above was enriched in the nuclear fractions of NRVMs, consistent with the immunostaining data, further supporting the localization of the C-terminus of Ca_v_1.3 in the nucleus of cardiac myocytes (Figure 1L). Adequate fractionation quality was verified using Histone Deacetylase antibody (HDAC), confirming minimal cross-contamination between the nuclear and cytoplasmic fractions.

Ca_v_1.3 gene undergoes alternative splicing yielding Ca_v_1.3 α1 subunits with a long (usage of exon 42, Ca_v_1.342) or short (usage of exon 42A, Ca_v_1.342) C-terminus [21]. The longer form of the C-terminus has been shown to contain several regulatory protein–protein interactions as well as regulatory motifs common to the entire Cav1.x family [21]. A blast search shows that Ca_v_1.3 shares more than 70% homology with Ca_v_1.2. Comparison of the aligned amino acid sequences around the site of proteolytic cleavage reveals a striking conservation of amino acid sequence between Ca_v_1.2 and Ca_v_1.3 [41]. Although the exact proteolytic cleavage site on Ca_v_1.3 is not known, the high level of amino acid sequence conservation is consistent with the conclusion that Ca_v_1.3 channels are potentially cleaved at the equivalent site around amino acid 1700 (Figure S2).

Collectively, these data support the premise that the C-terminus of Ca_v_1.3 is cleaved from the full-length protein and subsequently translocates to the nucleus.

### 3.2. The Expression of the Truncated Ca_v_1.3-C-Terminus in NRVMs Translocates to the Nucleus of Transfected Cells and Modulates the Transcription of Endogenous Genes

**Figure 2:**
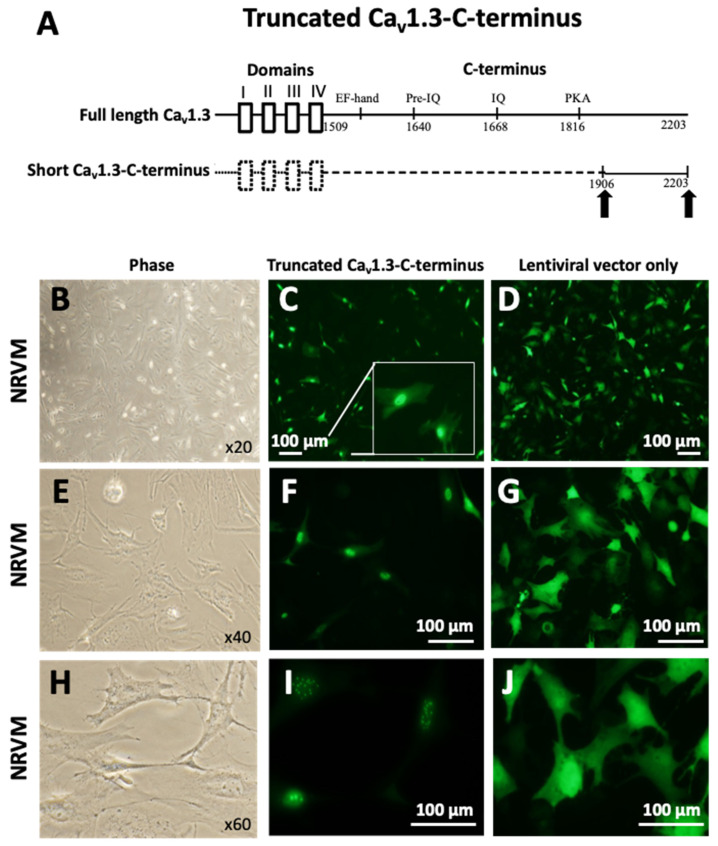
The truncated Ca_v_1.3-C-terminus expressed in NRVMs translocates to the nucleus. (**A**) Schematic representation of wild-type full-length Ca_v_1.3 and the short Ca_v_1.3-C-terminus construct containing amino acids 1906–2203 indicated by arrows. Microscope observation of NRVMs in phase view (magnification ×20) (**B**), transfected with Ca_v_1.3-C-terminus-GFP (pLVX-GFP1-a1DCa_v_1.3) (**C**) and transfected with lentiviral vector-GFP (pLVX-GFP1-N1) only (**D**). Microscope observation of NRVMs in phase view (magnification ×40) (**E**) transfected with Ca_v_1.3-C-terminus-GFP (**F**) and transfected with lentiviral vector-GFP only (**G**). Microscope observation of NRVMs in phase view (magnification ×60) (**H**) transfected with Ca_v_1.3-C-terminus-GFP (**I**) and transfected with lentiviral vector-GFP only (**J**). The white box shows zoomed area. Panels B, E, and H show the phase views corresponding to panels C, F, and I, respectively. The scale bar is 100 µm.

To explore the role of the C-terminus in regulating the transcription of endogenous genes, we developed and tested a GFP-lentiviral vector incorporating a short distal Ca_v_1.3-C-terminus construct of 297 amino acids (Figure 2A), derived from its distal portion and including the 1906–2203 amino acids of the channel protein (Ca_v_1.3-C-terminus-GFP). The vector integration efficiency was determined by immunostaining for GFP upon infection of NRVMs with the same construct. NRVMs were plated at 60–70% confluence, infected on day 2, and cultured for 48 h. An infection rate approaching 100% was observed, highlighting the effectiveness of this method for delivery into cardiomyocytes (Figure 2B–D). Similar results have been previously reported by our group [35]. Upon transduction with both Ca_v_1.3-C-terminus-GFP and an empty vector GFP, we found that the C-terminus of Ca_v_1.3 translocates to the nucleus of NRVMs (Figure 2B–G). This was confirmed by the presence of distinct speckles within the nucleus of Ca_v_1.3-C-terminus-GFP-infected cells (Figure 2D,G). Indeed, a punctate or speckled staining pattern is a hallmark characteristic of interchromatin spaces where splicing factors are located [42].

**Figure 3:**
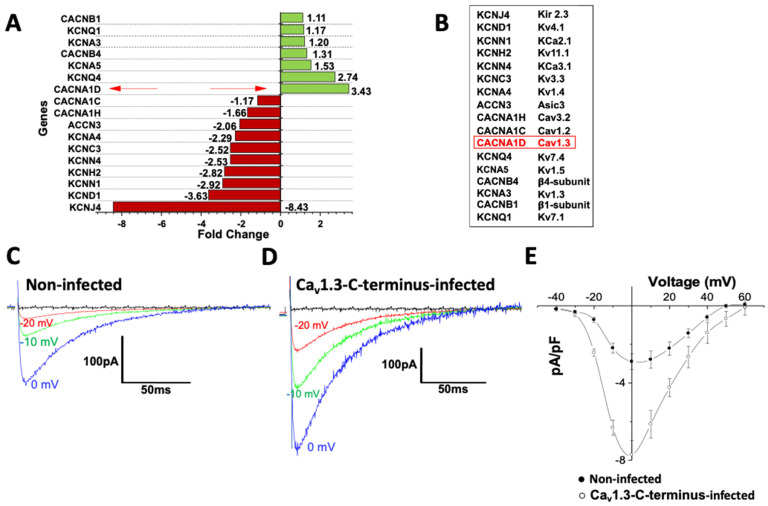
The expression of the truncated Ca_v_1.3-C-terminus in NRVMs modulates endogenous genes. (**A**) Targeted ion channel genes expression in NRVMs upon Ca_v_1.3-C-terminus-GFP construct injection relative to lentiviral-GFP vector. Green bars indicate upregulated genes, whereas red bars indicate downregulated genes. The red arrow indicates the Cav1.3 (CACNA1D) gene. (**B**) The inset displays the channel associated with each analyzed gene. The red boxindicates the Ca_v_1.3 (*CACNA1D*) gene. (**C**) Total L-type Ca^2+^ current recorded from non-infected and (**D**) Ca_v_1.3-C-terminus-GFP-infected single NRVMs. (**E**) The corresponding current–voltage relationships from non-infected (n = 6 cells) and Ca_v_1.3-C-terminus-GFP-infected NRVMs (n = 6 cells, from 3 different culture dishes).

A targeted RT^2^ Profiler PCR array was used to identify mRNAs that are transcriptionally regulated by the C-terminus expression. The NRVMs, treated with Ca_v_1.3-C-terminus-GFP or GFP-lentivirus alone, were assessed for the mRNA expression profiles of the targeted genes. A total of eighty-six genes were analyzed in triplicate. Genes were considered significantly regulated if they exhibited a fold change ≥ 2.0 or ≤−2.0 together with statistical significance (*p* < 0.05). Our findings show that, in NRVMs, transfection with the Ca_v_1.3-C-terminus-GFP induced a significant 3.43-fold increase in *CACNA1D* gene expression, followed by significant upregulation of additional ion channel genes, including *KCNQ4* (2.74-fold) and *KCNA5* (2.74-fold). In contrast, several genes were significantly downregulated, including *KCNJ4* (−8.43-fold), while *CACNA1C* (−1.17-fold) showed a non-significant trend toward downregulation. These results indicate that the Ca_v_1.3-C-terminus modulates the expression of multiple endogenous genes (Figure 3A,B). NRVMs infected with empty vector containing GFP-lentivirus did not affect the gene expression when compared with non-infected NRVMs.

**Figure 4:**
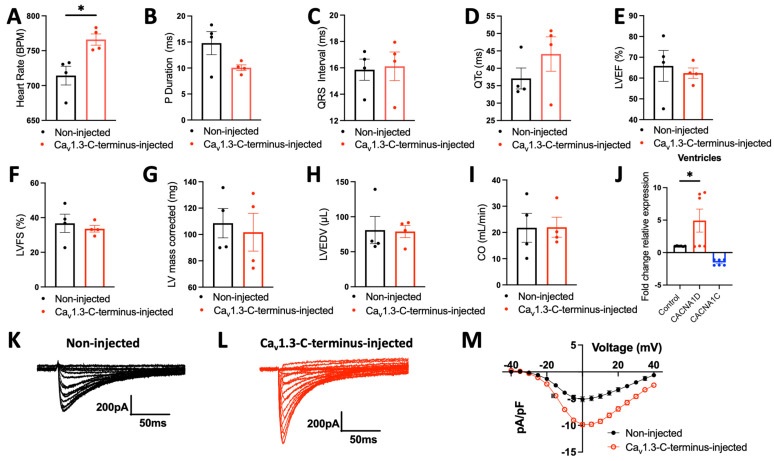
Ca_v_1.3-C-terminus injection had no major electrocardiographic and echocardiographic effects in mice. (**A**) Heart rate distribution between wild-type mice (WT, non-injected, black bar), and WT mice treated with 3 × 10^11^ GC/mL AAV9-Ca_v_1.3-C-terminus for 30 days (Ca_v_1.3-C-terminus-injected, red bar). * *p* < 0.05. (**B**) P duration, (**C**) QRS interval, and (**D**) QTc in the two groups. (**E**) Left ventricular (LV) ejection fraction (EF), (**F**) LV fractional shortening (FS), (**G**) LV mass corrected, (**H**) LV end-diastolic volume (EDV), and (**I**) cardiac output (CO) in WT non-injected mice and WT mice treated with 3 × 10^11^ GC/mL AAV9-Ca_v_1.3-C-terminus for 30 days. The number of experiments is based on 4 mice (equal sex distribution) aged between 7 and 8 months old for each parameter. The data are presented as mean ± SEM. Significance was calculated using a paired t-test. (**J**) RT-qPCR of calcium voltage-gated channel subunit alpha1 D (*CACNA1D,* red bar), and calcium voltage-gated channel subunit alpha1 C (*CACNA1C,* Blue bar) gene expression in ventricles of mice injected with 3 × 10^11^ GC/mL AAV9-Ca_v_1.3-C-terminus. Fold-change expression was calculated relative to control (black bar). The number of experiments is based on 6 independent replicates. The data are shown as mean ± SEM. Significance was calculated using one-way ANOVA and Tukey’s post hoc test. * *p* < 0.05. (**K**) Total L-type Ca^2+^ current recorded from non-injected WT and (**L**) Ca_v_1.3-C-terminus-injected WT mice treated with 3 × 10^11^ GC/mL AAV9-Ca_v_1.3-C-terminus for 30 days. (**M**) The corresponding current–voltage relationships from non-injected (n = 9 cells) and Ca_v_1.3-C-terminus-injected WT mice (n = 10 cells, from 3 different mice).

Total L-type Ca^2+^ current (I_CaL_) was recorded from non-infected and Ca_v_1.3-C-terminus-GFP-infected single NRVMs. The results are shown in Figure 3C–E, where I_CaL_ current density was significantly (*p* < 0.01) increased from 3.2 ± 0.47 pA/pF in non-infected single NRVMs to 7.8 ± 0.35 pA/pF in Ca_v_1.3-C-terminus-GFP-infected NRVMs, suggesting that Ca_v_1.3 gene upregulation translated to a functional increase in the total I_CaL_ density (not due to Ca_v_1.2 since it was downregulated, see Figure 3A and Figure 4J).

To assess the impact of the Ca_v_1.3-C-terminus as a potential candidate for future gene therapy applications in cardiovascular diseases, we injected in vivo wild-type (WT) mice with 3 × 10^11^ GC/mL Ca_v_1.3-C-terminus-GFP adeno-associated virus 9 (AAV9-Ca_v_1.3-C-terminus) intraperitoneally for 30 days. AAV9 is known to exhibit strong cardiomyocyte tropism in vivo, resulting in preferential and efficient gene transfer to cardiac tissue after systemic delivery [43,44,45]. Consistent with this, multiple studies have shown that a single AAV9 administration is sufficient to achieve robust and long-lasting cardiac expression, with efficient cardiomyocyte transduction following a one-time injection [23,46,47,48,49]. Electrocardiogram (ECG) recordings were performed using a paired experimental design in which each mouse served as its own control, with measurements obtained at baseline (prior to AAV9-Ca_v_1.3-C-terminus injection) and 30 days post-AAV9-Ca_v_1.3-C-terminus injection to assess cardiac electrical changes induced by gene delivery. AAV9-Ca_v_1.3-C-terminus injection increased heart rate in mice (Figure 4A) but did not affect the other ECG parameters, including P- wave duration, QRS interval or heart rate-corrected QT interval (QTc), all of which remained unchanged (Figure 4B–D). To evaluate any potential AAV9-Ca_v_1.3-C-terminus injection-induced cardiac alterations, we assessed the hemodynamic parameters using echocardiography (Echo). We found no significant differences between injected and non-injected groups for the left ventricular (LV) ejection fraction (LVEF), LV fractional shortening (LVFS), LV mass corrected, LV end-diastolic volume (LVEDV), and cardiac output (CO) confirming preserved cardiac structure and function and supporting the cardiac safety of the AAV9-Ca_v_1.3-C-terminus (Figure 4E–I). Notably, the adult human ventricles do not express *CACNA1D* [37]. Moreover, Mangoni and collaborators have previously shown the absence of Ca_v_1.3 in the adult ventricle of mice [8]. We found increased levels of *CACNA1D* gene expression (+4.9-fold) and decreased levels of *CACNA1C* (1.5-fold) in the ventricles of mice upon AAV9-Ca_v_1.3-C-terminus injection (Figure 4J). The results we observed in vitro were thus corroborated by our in vivo findings.

Total I_CaL_ was recorded from non-injected WT and Ca_v_1.3-C-terminus-injected WT mice treated with 3 × 10^11^ GC/mL AAV9-Ca_v_1.3-C-terminus for 30 days. The results are shown in Figure 4K–M, where I_CaL_ current density was significantly (*p* < 0.0001) increased from 5.1 ± 0.47 pA/pF in non-injected WT mice to 9.8 ± 0.41 pA/pF in Ca_v_1.3-C-terminus-injected WT mice, suggesting that Ca_v_1.3 gene upregulation translated to a functional increase in the total I_CaL_ density. This finding is consistent with the increased I_CaL_ current density observed in Ca_v_1.3-C-terminus-GFP-infected NRVMs and with our RT-qPCR results demonstrating elevated *CACNA1D* mRNA expression.

### 3.3. Promoter Constructs of the Ca_v_1.3 Promoter

**Figure 5:**
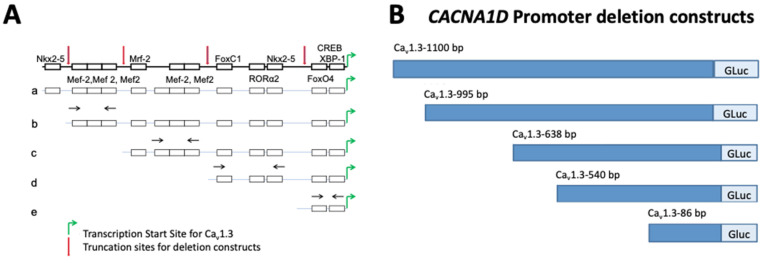
Schematic construction of Ca_v_1.3 promoter. (**A**) Schematic diagram of the Ca_v_1.3 promoter showing different putative binding sites for various transcription factors and truncation sites; red arrows show the localization of the cut for each construct (1100, 995, 638, 540, and 86 bp). Green arrows indicate the transcription start site for the Ca_v_1.3 promoter. Black arrows show the primer sets used to amplify potential interaction sites for the C-terminal domain. (**B**) Ca_v_1.3 (*CACNA1D*) promoter deletion constructs made by sequential N-terminal deletion using 5 forward and 5 reverse primers leading to subsequent deletion of MEF2 transcription factor binding sites.

Ca_v_1.3 promoter (accession number NM_028981.2) was used for the promoter experiments. The promoter of Ca_v_1.3 was identified as the region ~1100 bp upstream of the transcription start site (TSS) of Ca_v_1.3. The TSS was determined using the database of transcription start sites (DBTSS). DBTSS contains experimentally determined information on positions of the TSS of numerous genes. This information was further verified using Genomatix software suite (Genomatix Software GmbH, accessed in 2021). Further bioinformatic analysis using ConSite and Transfac (geneXplain GmbH, accessed in 2021) revealed that the promoter region of Ca_v_1.3 consists of numerous common transcription factor recognition sites as shown in Figure 5A. To determine how the Ca_v_1.3-C-terminus auto-regulates promoter activity, we designed different truncation deletion sites to identify the *cis*-elements required for C-terminus peptide activity (Figure 5A,B). This will enable the identification of actors involved in both positive and negative regulation of the promoter. These constructs were created with sequential deletion of the MEF2 transcription factor binding sites on the Ca_v_1.3 promoter (Figure 5A,B).

**Figure 6:**
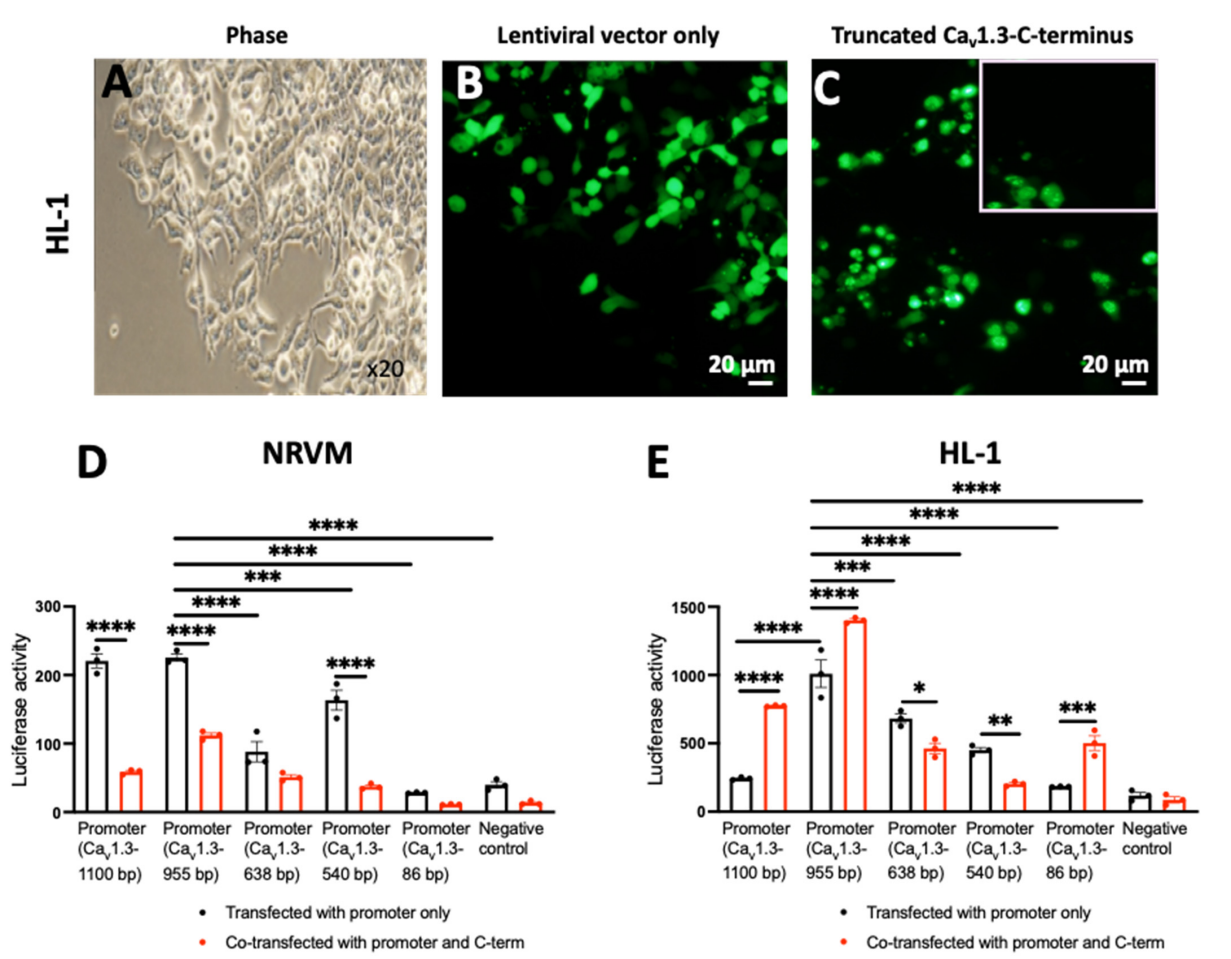
Luciferase assay in NRVMs and HL-1 cardiomyocytes transfected with Ca_v_1.3 N-terminal deletion promoter constructs in pEZX-PG02 vector only or co-transfected with Ca_v_1.3-C-terminus. Microscope observation of mouse atrial cardiomyocytes (HL-1) in phase view (magnification ×20) (**A**), transfected with lentiviral vector GFP (control, pLVX-GFP1-N1) only (**B**) and transfected with Ca_v_1.3-C-terminus-GFP (pLVX-GFP1-a1DCa_v_1.3) (**C**). The white box shows zoomed area. The scale bar is 20 µm. Luciferase activity measured in NRVMs (**D**) and HL-1 cardiomyocytes (**E**) transfected with the promoter constructs (black bars) or co-transfected with Ca_v_1.3 promoter constructs and the Ca_v_1.3-C-terminus (red bars). Experiments were conducted using 3 independent biological replicates. The data are shown as mean ± SEM. * *p* < 0.05, ** *p* < 0.01, *** *p* < 0.001, and **** *p* < 0.0001. Statistical significance was calculated using two-way ANOVA with Tukey’s multiple comparison test.

Upon transduction with both Ca_v_1.3-C-terminus-GFP and an empty vector GFP, we found that the C-terminus of Ca_v_1.3 also translocates to the nucleus of mouse atrial HL-1 cardiomyocytes (Figure 6A–C). NRVMs and HL-1 cardiomyocytes transfected with Ca_v_1.3 promoter N-terminal deletion constructs showed a gradual decrease in luciferase activity (from 225 ± 5.25 for Ca_v_1.3-955 bp promoter to 28.26 ± 0.48 for Ca_v_1.3-86 pb promoter in NRVMs, *p* < 0.0001; and from 1010 ± 101 for Ca_v_1.3-955 bp promoter to 183 ± 1.14 for Ca_v_1.3-86 pb promoter in HL-1, *p* < 0.0001) (Figure 6D,E). However, in NRVMs, the Ca_v_1.3-638 bp promoter showed lower luciferase activity than expected owing to the possible loss of an activator site (Figure 6D). In HL-1 cardiomyocytes, the luciferase activity of the full-length promoter (Ca_v_1.3-1100 bp) was 4-fold lower than that of the 955 bp (Ca_v_1.3-955 bp) deletion construct (Figure 6E). The progressive reduction in luciferase activity from the Ca_v_1.3-955 bp construct to the Cav1.3-86 bp construct suggests the presence of positive *cis*-regulatory elements, including activator binding sites, within the deleted regions.

### 3.4. MEF2 Binding Sites Contribute to Ca_v_1.3 Promoter Activation by the C-Terminus

To investigate the contribution of different elements to promoter regulation and determine the *cis*-elements essential for the C-terminus to modulate Ca_v_1.3 promoter activity, we co-transfected both NRVMs and HL-1 cardiomyocytes with both the Ca_v_1.3 promoter constructs and the Ca_v_1.3-C-terminus (Figure 6D,E). Co-transfection of NRVMs with the Ca_v_1.3 promoter constructs and Ca_v_1.3-C-terminus resulted in a consistent decline in luciferase activity (3.7-fold, 2-fold, 4.4-fold, and 2.5-fold for Ca_v_1.3-1100 bp promoter, Ca_v_1.3-955 bp promoter, Ca_v_1.3-540 bp promoter, and Ca_v_1.3-86 bp promoter, respectively) (Figure 6D). In HL-1 cardiomyocytes, we found an increase in luciferase activity of 4-fold, 1.4-fold, and 2.7-fold of the full-length Ca_v_1.3-1100 bp promoter, Ca_v_1.3-955 bp and Ca_v_1.3-86 bp promoter, respectively, in the presence of the Ca_v_1.3-C-terminus (Figure 6E). However, the Ca_v_1.3-638 bp and Ca_v_1.3-540 bp promoters showed a decrease in luciferase activity (1.5- and 2-fold, respectively). Interestingly, the Ca_v_1.3-C-terminus co-transfected into NRVMs and HL-1 cardiomyocytes with the Ca_v_1.3 promoter variant NM_001083616, a shorter form of the gene, resulted in increased luciferase activity (Figure S3). Deleting the MEF2 binding sites on the promoter consistently impacted its regulation by the Ca_v_1.3-C-terminus. This suggests that the Ca_v_1.3-C-terminus may regulate the Ca_v_1.3 promoter through mechanisms involving MEF2-dependent transcription.

## 4. Discussion

In the present work, we demonstrated that the Ca_v_1.3-C-terminus (1906–2203) translocates to the nucleus of NRVMs and HL-1 cardiomyocytes. This was confirmed by both the presence of nuclear speckles in the transfected cells and by the Ca_v_1.3-C-terminus antibody that predominantly stained the nucleus and perinuclear region and, to a lesser extent, the cytoplasm in both NRVMs and ARAMs. Moreover, the Western blot and confocal imaging data, combined with the detection of the C-terminus in the nuclear fraction, together support the premise that the C-terminus of Ca_v_1.3 is mobile, and translocates to the nucleus. In fact, the C-terminal region of the Ca_v_1.3 channel is known to be under extensive alternative splicing, which can significantly affect the channel function [21]. This is likely attributed to the gating role of the C-terminus, which helps prevent Ca^2+^-dependent inactivation of the channel [50].

Furthermore, the Ca_v_1.3-C-terminus in vitro in NRVMs, and in vivo in mice hearts led to altered expression of both Ca^2+^ channel genes, the *CACNA1D*, and the *CACNA1C*. These findings align with observations in Ca_v_1.2 and Ca_v_1.3 channels reported in previous investigations by other groups [14,16]. The cleavage of the C-terminus of L-type Ca^2+^ channels was first reported in the Ca_v_1.2 channel. It has been previously demonstrated that, in neuronal cells of the rat brain, the Ca_v_1.2 C-terminus is cleaved and translocates to the nucleus, where it regulates the transcription of various genes [14]. In 2013, this same group demonstrated that the C-terminus transcriptional regulator is produced by the activation of a cryptic promoter in exon 46 of the Ca_v_1.2 gene [15]. In cardiomyocytes, the C-terminus of Ca_v_1.2 has also been shown to exert auto-inhibitory effects on Ca_v_1.2 channel function [41]. It further reduces the basal luciferase activity of the mouse Ca_v_1.2 promoter [51] along with Ca_v_1.2 channel activity [41].

Interestingly, NRVMs treated with Ca_v_1.3-C-terminus-GFP displayed a 3.43-fold increase in *CACNA1D* gene expression level, which was the highest level of upregulation. This finding reveals a novel mechanism, as it demonstrates *for the first time* that the C-terminus fragment of Ca_v_1.3 serves as an auto-trans-regulator of its own gene expression. This contrasts with prior findings for Ca_v_1.2, where C-terminus acts as an auto-repressor [51], highlighting a distinct regulatory mechanism of gene expression specific to Ca_v_1.3. Another gene that was upregulated is *KCNQ4* (2.74-fold), a voltage-dependent K channel-Kv7.4, which is strongly expressed in inner ear cells but nearly undetectable in non-infected control NRVMs, and *KCNA5* (2.74-fold), a Kv1.5 delayed rectifier. Introducing the C-terminus fragment into cultured NRVMs led to the regulation of specific genes critical for cell excitability. This phenomenon is not uncommon, as previous studies have demonstrated that the C-terminus of Ca_v_1.2 not only regulates its own gene expression but also influences the expression of other genes [14]. However, the underlying molecular mechanisms remain to be fully elucidated.

We have previously demonstrated that the adult human ventricles lack *CACNA1D* expression [37]. Here, we found that the Ca_v_1.3-C-terminus promotes Ca^2+^ channel expression by enhancing expression of its own gene, *CACNA1D*, while repressing *CACNA1C* expression. Likewise, in vivo administration of AAV9-Ca_v_1.3-C-terminus in mice resulted in an approximately 4.9-fold upregulation of *CACNA1D* in the ventricles. This is in line with our in vitro results wherein in NRVMs (ventricular) we observed an upregulation of the Ca_v_1.3 gene. The Ca_v_1.3-C-terminus also increased total L-type Ca^2+^ current density in NRVMs and in mice injected with AAV9-Ca_v_1.3-C-terminus. L-type Ca^2+^ channels are composed of several subunits and the beta subunit regulates channel kinetics and increases their open probability [52]. Thus the increased current could be due to increased L-type Ca^2+^ current density as well as to increased beta4 and beta1 subunits encoded by *CACNB4* and *CACNB1,* respectively, as demonstrated by Colecraft et al. in 2002 [52]. Collectively, these results indicate that the Ca_v_1.3-C-terminus differentially regulates Ca^2+^ channel gene expression, promoting or reducing transcription depending on the gene. This is likely due to differences in the accessory proteins which bind to the promoter in each gene. A similar scenario has been previously reported in which a gene transitions from functioning as an activator to acting as a repressor [53,54]. Our data indicate that the effect of the Ca_v_1.3-C-terminus on Ca^2+^ channels gene transcription varies in a gene-specific manner. However, further evaluation is required to elucidate the mechanism by which the Ca_v_1.3-C-terminus regulates Ca_v_1.3 gene expression in atrial and ventricular cells. ECG and Echo recordings were performed at baseline (prior to AAV9-Ca_v_1.3-C-terminus injection) and 30 days post-AAV9-Ca_v_1.3-C-terminus injection to assess cardiac electrical changes and hemodynamic parameters alterations induced by gene delivery. AAV9-Ca_v_1.3-C-terminus injection increased heart rate in mice but did not affect any of the other evaluated parameters, confirming preserved cardiac structure and function and supporting the cardiac safety of the AAV9-Ca_v_1.3-C-terminus. This could potentially lead to a novel therapeutic approach using gene editing to restore Ca^2+^ defects in certain cardiac diseases, like Sinus Node Dysfunction and Deafness Syndrome [55,56,57], heart failure [23], Brugada syndrome [58,59], short QT syndrome [60], and early repolarization syndrome [61,62] where additional Ca^2+^ entry is desirable.

While conventionally, a significant increase in heart rate is expected to change the PR and QT intervals, AAV9-mediated Ca_v_1.3-C-terminus expression may selectively increase heart rate, reflected by a shortened RR interval, without significant changes in PR, QRS, or QT intervals. This pattern is consistent with a primary chronotropic effect on sinoatrial node automaticity with minimal impact on atrioventricular conduction, ventricular depolarization, or repolarization.

To better assess Ca_v_1.3-C-terminus’s ability to modify the transcription of Ca^2+^ channel gene, Ca_v_1.3, we created a series of deletion constructs of the Ca_v_1.3 promoter. Bioinformatics analysis using ConSite transcription factor binding site prediction software showed that the region from 1100 to 955 bp upstream of the transcription start site consists of many putative transcription factor binding sites, including the MEF2 binding site.

We sequentially deleted N-terminal regions from the promoter which simultaneously led to the deletion of MEF2 transcription factor binding sites. MEF2 regulates the expression of many cardiac structural and contractile genes [63] and therefore could play an important role in the regulation of the Ca_v_1.3 promoter. The Ca_v_1.3-955 bp construct contains three MEF2 binding sites, the Ca_v_1.3-638 bp contains two MEF2 binding sites, while the Ca_v_1.3-540 bp contains just one MEF2 binding site. Ca_v_1.3-86 bp contains no MEF2 binding sites. A similar study by Schroder et al. [51] has previously shown that the Ca_v_1.2-C-terminus interacts with the Ca_v_1.2 promoter in adult rabbit ventricular myocytes at Nkx2.5, MEF2 and C/EBP binding sites and that overexpression of the Ca_v_1.2-C-terminus decreased luciferase activity of the promoter. Here, in NRVMs, we found a gradual decrease in luciferase activity with sequential deletion of N-terminal sequences of the Ca_v_1.3 promoter, except for Ca_v_1.3-540 bp that showed increased luciferase activity. These results suggest that the region from 638 bp to 540 bp, located upstream of the transcription start site, contains one or more repressor binding sites, the removal of which leads to an increase in promoter activity. Likewise, we found a consistent and gradual decrease in luciferase activity with sequential deletion of N-terminal sequences from Ca_v_1.3-955 bp to Ca_v_1.3-86 bp in HL-1 cardiomyocytes. Thus, the binding sites within all three constructs, Ca_v_1.3-955 bp, Ca_v_1.3-638 bp and Ca_v_1.3-540 bp, collectively contributed to the optimal basal promoter activity. Interestingly, in HL-1 cardiomyocytes, the promoter activity of the full-length promoter (Ca_v_1.3-1100 bp) was not significantly higher than that of the negative control. In contrast, the basal promoter activity of Ca_v_1.3-955 was 5-fold greater than the negative control. It is therefore likely that the region of the 1100–955 bp promoter upstream of the Ca_v_1.3 TSS contains a repressor binding site. Transfection of Ca_v_1.3-86 bp into HL-1 cardiomyocytes and NRVMs resulted in the absence of basal promoter activity. This suggests that the elements responsible for basal promoter activity are likely found within 955–86 bp, upstream of the transcription start site of Ca_v_1.3.

The effect of the Ca_v_1.3-C-terminus on the regulation of Ca_v_1.3 promoter deletion constructs was further explored by co-transfecting the Ca_v_1.3-C-terminus with the Ca_v_1.3 promoter constructs into HL-1 cardiomyocytes and NRVMs. We found that the Ca_v_1.3-C-terminus reduced luciferase activity across all promoter constructs in NRVMs, suggesting a repressive effect under these conditions. Interestingly, we observed that co-transfection of the Ca_v_1.3-C-terminus into NRVMs with the Ca_v_1.3 promoter variant NM_001083616, corresponding to the shorter-length gene form, led to an increase in luciferase activity. These findings suggest that the C-terminus may exert differential regulatory effects depending on the promoter context or construct used. Alternatively, the Ca_v_1.3-C-terminus could modulate transcription through interactions with transcriptional co-regulators, influencing its regulatory function.

The most pronounced effect of C-terminus binding in co-transfected HL-1 cardiomyocytes is observed with the Ca_v_1.3-1100 bp promoter, wherein a 4-fold upregulation in activity is detected. This suggests that the Ca_v_1.3-C-terminus binds preferentially to the upstream regulatory regions. Overall, our results suggest that MEF2, NKX2.5, FOXC1, and RORa2 act as activators of Ca_v_1.3 transcription, leading to increased expression. Unexpectedly, the two MEF2 binding sites located between 638 bp and 540 pb are predicted to exert repressive effects. In addition, the lack of luciferase activity in the 86 bp construct indicates that CREB and XBP1 are also likely to function as transcriptional repressors of Ca_v_1.3.

Overall, our results from Ca_v_1.3-C-terminus transfection in NRVMs demonstrate an increase in Ca_v_1.3 mRNA levels. However, when the Ca_v_1.3-C-terminus is co-transfected with the Ca_v_1.3 promoter constructs, the opposite effect is observed, with promoter activity decreasing in NRVMs and increasing in HL-1 cardiomyocytes. One possible explanation for this discrepancy is that the endogenous regulatory regions in HL-1 cardiomyocytes and NRVMs may contain distinct *cis*-regulatory elements, including distal enhancers and activator or repressor binding sites, which are absent from the in vitro reporter constructs. Although our electrophysiological data using patch clamp technique showed upregulation of the total L-type Ca^2+^ current, the luciferase activity results are rather inconsistent, demonstrating a decrease in the promoter activity. The observed reduction in promoter activity alongside increased Ca_v_1.3 gene transcription in NRVMs is not uncommon and may be attributed to limitations of promoter–reporter assays. These constructs contain only partial promoter sequences that lack distal enhancers greater than 50 kb, native chromatin architecture, higher-order genomic interactions, and alternative promoter contributions that are critical for physiological gene regulation. Consequently, reporter activity may not accurately reflect endogenous transcription, particularly in complex and cell-type-specific environments. The activation observed with the shorter Ca_v_1.3 promoter variant NM_001083616 further supports a model of promoter length-dependent regulation, in which longer constructs may contain repressor elements absent in core promoters. Together, these findings suggest that the Ca_v_1.3-C-terminus exerts context-dependent regulatory effects and can act as a repressor or activator depending on promoter composition, cellular environment, and interacting cofactors. This pattern, involving repression of isolated canonical promoter constructs alongside activation of alternative promoters and net endogenous upregulation, is consistent with the complexity of cardiac gene regulation. In HL-1 atrial cells, abundant cardiac transcription factors favor activation of the same promoters. Thus, while luciferase assays define promoter regions responsive to the Ca_v_1.3-C-terminus, the combined RT-qPCR and electrophysiological data more accurately reflect its biological role as an endogenous trans-regulator of Ca_v_1.3 expression.

Moreover, while the Ca_v_1.3-C-terminus consistently increased endogenous *CACNA1D* expression and I_CaL_ current density, luciferase assays revealed cell-type-specific effects on isolated promoter fragments. Together with prior studies demonstrating nuclear signaling by L-type channel C-terminal fragments [14,16], these findings support a model in which the Ca_v_1.3-C-terminus acts as a context-dependent transcriptional regulator via interaction with nuclear cofactors, rather than as a direct DNA-binding transcription factor.

Finally, MEF2 represents a biologically plausible cofactor given its established role in Ca^2+^-dependent cardiac gene regulation [64,65], and prior studies have shown that mutation or deletion of MEF2 binding sites reduces cardiac promoter activity; however, its involvement remains putative in our study. Our deletion analysis indicates that regions containing predicted MEF2 binding sites contribute to Ca_v_1.3-C-terminus-dependent promoter responsiveness, but do not establish a direct mechanistic link. Importantly, while luciferase assays demonstrate functional promoter responsiveness, they do not provide evidence for direct DNA binding by the Ca_v_1.3-C-terminus, which is more likely to regulate transcription through interactions within nuclear protein complexes, as described for L-type channel C-terminal fragments [14].

## 5. Conclusions and Study Limitations

We conclude that: (1) The C-terminus of Ca_v_1.3 translocates to the nucleus of atrial and ventricular cells. (2) Once in the nucleus, the C-terminus modulates the transcriptional activity of the Ca^2+^ channel genes of Ca_v_1.3 (upregulation) and Ca_v_1.2 (downregulation). (3) The C-terminus regulates Ca_v_1.3 promoter activity. (4) The C-terminus may regulate promoter activity through mechanisms involving MEF2-dependent transcription. Leveraging the unique intrinsic properties of the C-terminus as a transcriptional trans-regulator of its own gene expression may represent a potential approach to modulate cardiac function and warrants further investigation for therapeutic applications. While our findings have important translational impact, the potential impact of Ca_v_1.3-C-terminus overexpression on cardiac excitation–contraction coupling, along with its influence on heart rate and myocardial contractility, warrants further investigation addressing the precise mechanism by which a transcription factor regulates the expression of genes beyond its own. Consequently, the impact of the Ca_v_1.3-C-terminus on the expression of various endogenous genes merits deeper exploration. The relative contribution of direct transcriptional regulation by the Ca_v_1.3-C-terminus and indirect calcium-dependent signaling pathways cannot be distinguished, and therefore additional mechanistic studies will be required to resolve these distinct regulatory components. In the present study, we investigated a specific fragment of the Ca_v_1.3-C-terminus. In-depth analysis of the full-length Ca_v_1.3 channel, as well as additional C-terminal fragments, will be warranted to fully elucidate their respective roles and warrants future investigation. Moreover, the absence of in vivo analyses incorporating the different Ca_v_1.3-C-terminus cleavage forms (1100 bp, 540 bp, and 86 bp) remain to be explored. Future studies integrating these forms will be warranted to assess their responses to endogenous regulatory programs and their differential contributions to cardiac function, thereby further elucidating the intrinsic properties of the Ca_v_1.3-C-terminus regions. Also, it would be interesting to measure Ca_v_1.3 protein level because the beta4 subunit increased, which could increase the channel open probability. Given that Ca_v_1.3 is specifically expressed in the supraventricular tissue, it would be interesting to assess the impact of the Ca_v_1.3-C-terminus on atrial tissue and or the HL-1 cell line. While we compared the current densities of Ca_v_1.3-C-terminus-transduced neonatal myocytes with non-infected myocytes, we did not include mock-infected cells as an additional control. We did not assess direct physical interaction between the Ca_v_1.3-C-terminus and promoter DNA, as in experiments such as chromatin immunoprecipitation (ChIP), or perform site-directed mutagenesis of candidate MEF2 binding sites; therefore, the mechanism of transcriptional regulation, including the role of MEF2 and whether it is direct or indirect, remains to be determined. The effect of other transcription factors, such as NKX2.5, FOXC1, RORa2, CREB, and XBP1, on Ca_v_1.3 promoter activity warrant further investigations. It would also be valuable to assess the impact of the Ca_v_1.3-C-terminus on Ca_v_1.3 expression in other tissues where Ca_v_1.3 is typically expressed, such as neurons and endocrine cells. Finally, the long-term safety and potential pro-arrhythmic consequences of Ca_v_1.3 upregulation were not evaluated.

## Figures and Tables

**Table 1:** Primer pairs for *CACNA1D* promoter deletion constructs.

Forward Primer (5′-3′)	Reverse Primer (5′-3′)	Length of Construct
TCT 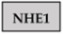 ACCCAGGGATRCTATGTGTTTAAGAA	ATTC 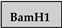 CACTTACAACTCTGCCTTTATATCGG	1100 bp
TCT 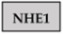 CCCCTAGGCACTGGACATAC	ATTC 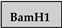 CACTTACAACTCTGCCTTTATATCGG	955 bp
TCT 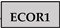 TCGGGAGGAAAAGCCAGTATG	ATTC 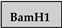 CACTTACAACTCTGCCTTTATATCGG	638 bp
TCT 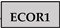 GCTTAGTAGGAGGGTGTCTGG	ATTC 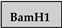 CACTTACAACTCTGCCTTTATATCGG	540 bp
TCT 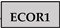 GCCAACAGCCACCTACCTAC	ATTC 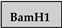 CACTTACAACTCTGCCTTTATATCGG	86 bp

## Data Availability

The datasets presented in this study are available from the corresponding author upon reasonable request.

## Supplementary Materials

The following supporting information can be downloaded at https://www.mdpi.com/article/10.3390/cells15090828/s1, Figure S1: The custom-made Ca_v_1.3-C-terminus antibody is specific; Figure S2: Conserved amino acid sequences of Ca_v_1.2 and Ca_v_1.3 channels at the sites of C-terminal interaction and proteolytic cleavage; Figure S3: Luciferase assay in NRVMs and HL-1 cardiomyocytes transfected with Ca_v_1.3 promoter variant NM_001083616 in pEZX-PG02 vector only or co-transfected with Ca_v_1.3-C-terminus.

## Non-Standard Abbreviations and Acronyms

The following abbreviations are used in this manuscript:

ARAMs	Adult Rat Atrial Myocytes
Ca^2+^	Calcium
SK2	Ca^2+^-activated potassium (K^+^) channels
NRVMs	Neonate Rat Ventricular Myocytes
SAN	Sino-Atrial Node
