# Supplementary material for: C-Terminus of Cav1.3 L-Type Ca2+ Channel Upregulates Its Own Gene Expression
Source: Cells. 2026 May 1;15(9):828. doi: 10.3390/cells15090828 (PMC13162661; doi:10.3390/cells15090828)
Supplement: Supplementary file 1 [file cells-15-00828-s001.zip › cells-4175318-supplementary.pdf]

Supplementary Figures

Figure S1

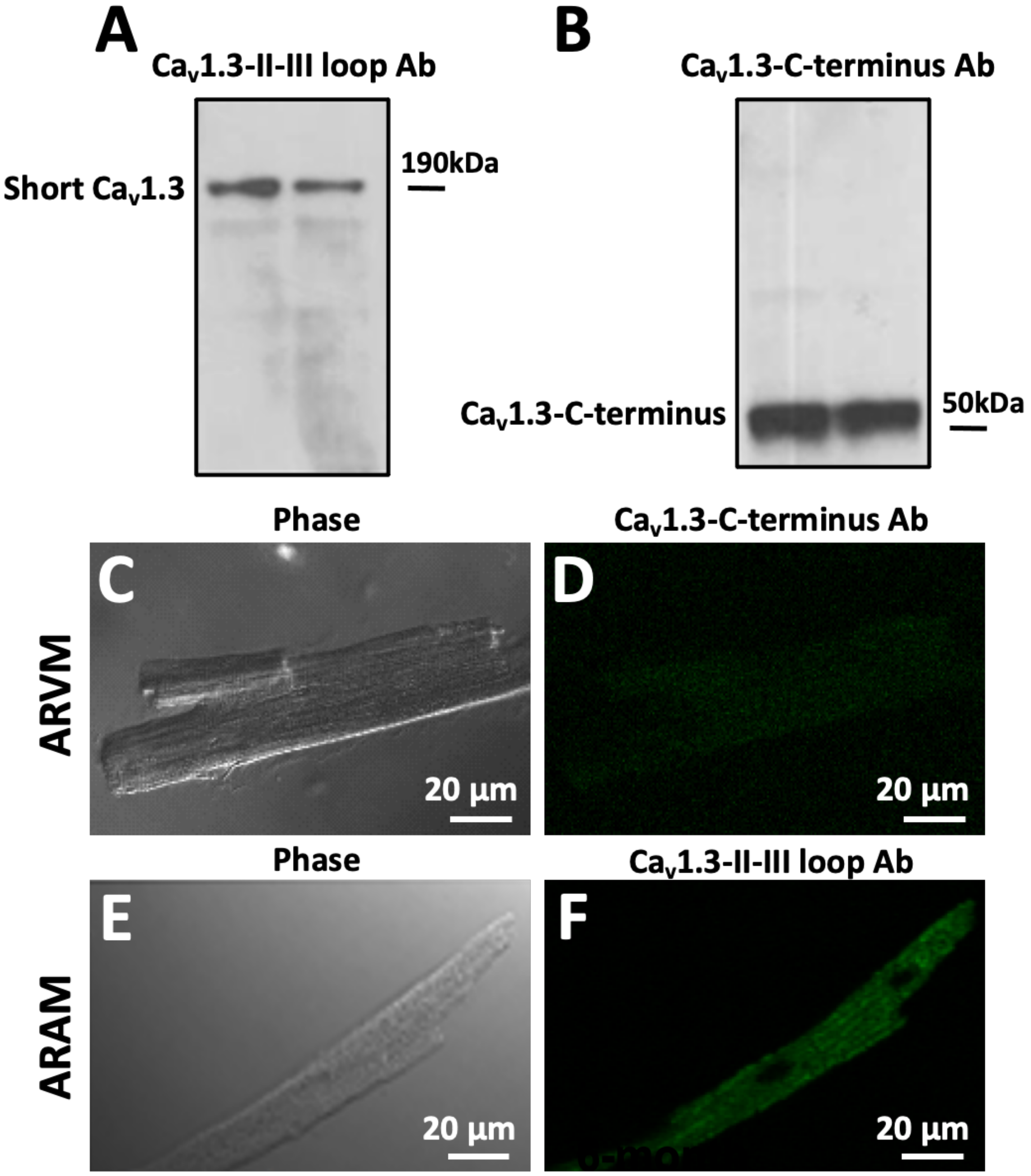

Figure S1: The custom-made $Ca_v1.3$-C-terminus antibody is specific. Western blot of $Ca_v1.3$ from adult mice atrial tissue probed with (A) anti-$Ca_v1.3$-II-III loop antibody and (B) anti-$Ca_v1.3$-C-terminus antibody. Experiments were done in duplicates. Ab=Antibody. (C) Phase view and (D) anti-$Ca_v1.3$-C-terminus antibody labeling in non-infected adult rat ventricular myocyte (ARVM). The scale bar is 20 µm. (E) Phase view and (F) anti-$Ca_v1.3$-II-III loop antibody labeling in non-infected adult rat atrial myocyte (ARAM). The scale bar is 20 µm.

Figure S2

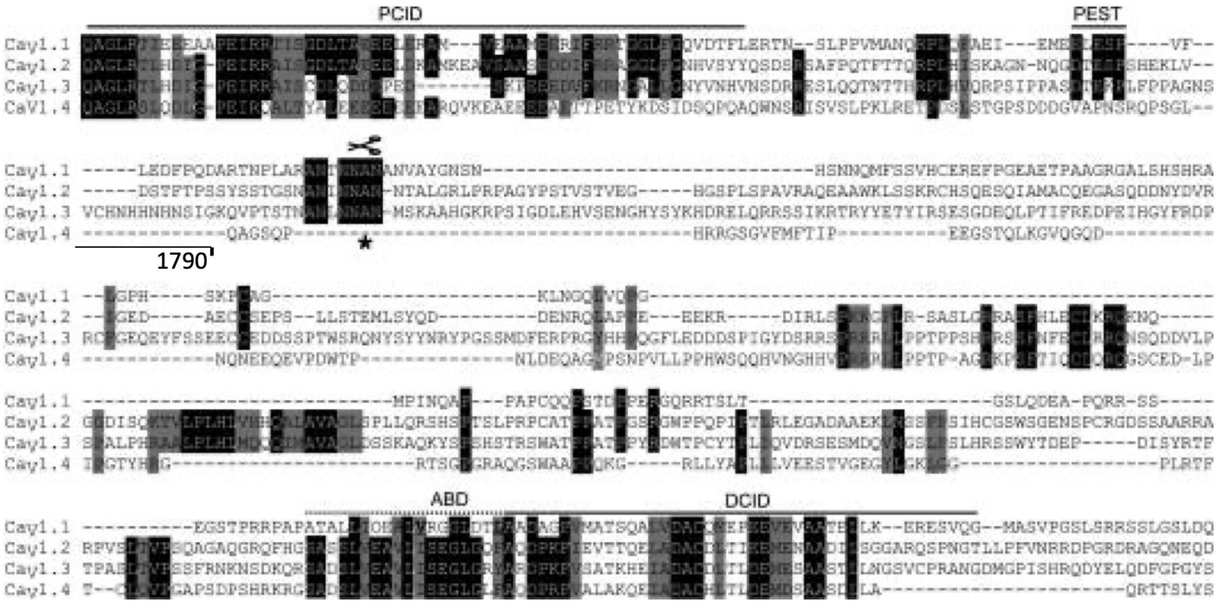

Figure S2: Conserved amino acid sequences of Ca$_v$1.2 and Ca$_v$1.3 channels at the sites of C-terminal interaction and proteolytic cleavage. Amino acid sequence alignment of the PCID, DCID, PEST motif, the proteolytic cleavage site (asterisk and scissors), and the AKAP15-binding domain (ABD; critical residues shown in bold) identified in Ca$_v$1.1 with Ca$_v$1.2, Ca$_v$1.3, and Ca$_v$1.4. Conserved amino acids are shaded in black, and similar amino acids are shaded in gray (adapted from [1]).

Figure S3

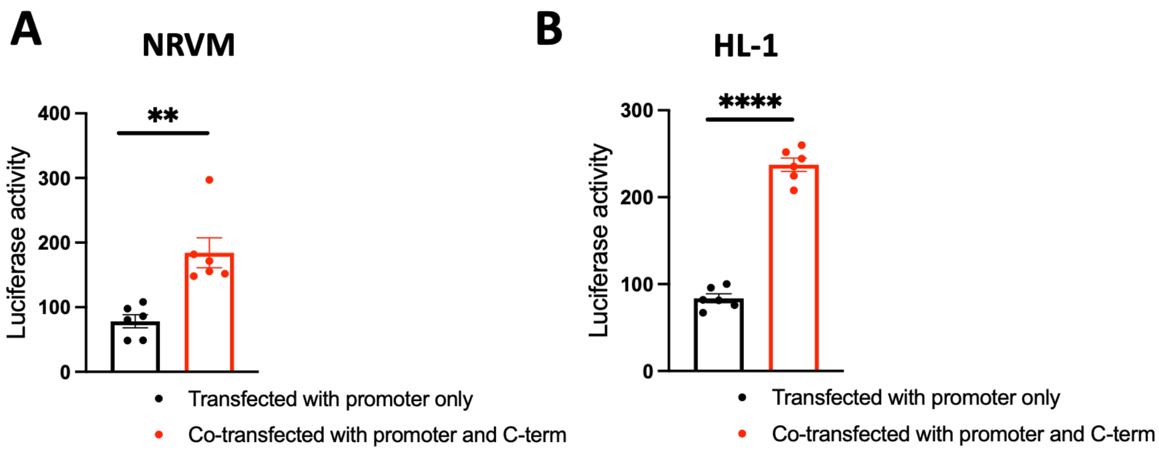

Figure S3: Luciferase assay in NRVMs and HL-1 cardiomyocytes transfected with Ca_v1.3 promoter variant NM_001083616 in pEZX-PG02 vector only or co-transfected with Ca_v1.3-C-terminus. Luciferase activity measured in neonatal rat myocyte (NRVMs) (A) and HL-1 cardiomyocytes (B) transfected with the Ca_v1.3 promoter variant NM_001083616 only (black bars and dots) or co-transfected with Ca_v1.3 promoter variant NM_001083616 with Ca_v1.3-C-terminus (red bars and dots). Experiments were conducted using 6 independent biological replicates. Data are shown as mean ± SEM. ** $p < 0.01$, and **** $p < 0.0001$. Statistical significance was calculated using Unpaired t-test.

Reference

1. Singh, A.; Gebhart, M.; Fritsch, R.; Sinnegger-Brauns, M.J.; Poggiani, C.; Hoda, J.-C.; Engel, J.; Romanin, C.; Striessnig, J.; Koschak, A. Modulation of Voltage- and Ca2+-Dependent Gating of CaV1.3 L-Type Calcium Channels by Alternative Splicing of a C-Terminal Regulatory Domain. *J Biol Chem* 2008, *283*, 20733–20744, doi:10.1074/jbc.M802254200.
